# Over-triage occurs when considering the patient's pain in Korean Triage and Acuity Scale (KTAS)

**DOI:** 10.1371/journal.pone.0216519

**Published:** 2019-05-09

**Authors:** Ji Hwan Lee, Yoo Seok Park, In Cheol Park, Hak Soo Lee, Ji Hoon Kim, Joon Min Park, Sung Phil Chung, Min Joung Kim

**Affiliations:** 1 Department of Emergency Medicine, Yonsei University College of Medicine, Seoul, Republic of Korea; 2 Emergency Medicine, College of Medicine, Kangwon National University Gangwondaehakgil, Chuncheon-si, Gangwon-do, Republic of Korea; 3 Department of Emergency Medicine, Inje University Ilsan Paik Hospital, Ilsanseo-gu, Goyang-si, Gyeonggi-do, Republic of Korea; University of Auckland, NEW ZEALAND

## Abstract

**Background:**

The Korean Triage and Acuity Scale (KTAS) was developed based on the Canadian Emergency Department Triage and Acuity Scale. In patients with pain, to determine the KTAS level, the pain scale is considered; however, since the degree of pain is subjective, this may affect the accuracy of KTAS. The purpose of this study was to evaluate the accuracy of KTAS in predicting patient's severity with the degree of pain used as a modifier.

**Method:**

A retrospective observational cohort study was conducted in an urban tertiary hospital emergency department (ED). We investigated patients over 16 years old from January to June 2016. The patients were divided into the pain and non-pain groups according to whether the degree of pain was used as a modifier or not. We compared the predictive power of KTAS on the urgency of patients between the two groups. Acute area registration in the ED, emergency procedure, emergency operation, hospitalization, intensive care unit admission, and 7-day mortality were used as markers to determine urgent patients.

**Results:**

Overall, 24,253 patients were included in the study, with 9,175 (37.8%) in the pain group. The proportions of patients with KTAS 1–3 were 61.4% in the pain and 75.6% in the non-pain groups. Among patients with KTAS 2–3, the proportion of urgent patients was higher in the non-pain group than the pain group (p<0.001). The odds ratios for urgent patients at each KTAS level revealed a more evident discriminatory power of KTAS for urgent patients in the non-pain group. The predictability of KTAS for urgent patients was higher in the non-pain group than the pain group (area under the curve; 0.736 vs. 0.765, p-value <0.001).

**Conclusions:**

Considering the degree of pain with KTAS led to overestimation of patient severity and had a negative impact on the predictability of KTAS for urgent patients.

## Introduction

Emergency department (ED) overcrowding has become a global trend due to an increased number of patients visiting the ED and boarding patients waiting for hospital admission.[1, 2] It is impossible to provide the best medical care quickly to all patients when the ED is overcrowded; therefore, early recognition of patients with life-threatening illnesses or injuries is very important.[3–5] Today, in most EDs, triage is performed to classify the severity of emergency patients within a few minutes of ED arrival. Accurate triage is important because under-triage may delay critical care of emergency patients while over-triage may inhibit the efficient management of ED resources.

Several countries have devised triage tools since the early 1990s. The Canadian Emergency Department Triage and Acuity Scale (CTAS), the Australian Triage Scale (ATS), the Emergency Severity Index (ESI), and the Manchester Triage Scale (MTS) based on the 5-level classification systems have greatly influenced triage in modern emergency rooms.[6–8] The CTAS was developed in 1999 by the Canadian Association of Emergency Physicians and National Emergency Nurses Affiliation, and revised in 2004 and 2008.[9, 10] The CTAS has been evaluated as a reliable tool and is being used in most emergency rooms in Canada today.[8, 11, 12] The CTAS has also been adopted by other countries such as Taiwan (Taiwan triage and acuity scale, TTAS) and Japan (Japanese triage and acuity scale, JTAS).[13, 14] In 2012, the Korean Triage and Acuity Scale (KTAS) was developed through the Ministry of Health and Welfare's research project.[15] The KTAS research team decided to introduce CTAS through a panel discussion of experts, and collected expert opinions using the Delphi method and supplemented KTAS with the Korean situation In 2015, KTAS training was conducted for medical staff in charge of triage of emergency rooms nationwide, and it was regulated by the government to use only KTAS in all domestic emergency rooms.

The KTAS consists of five acuity levels; from level 1 (resuscitation) to level 5 (non-urgent). Screening with the KTAS occurs first with the serious and life-threatening conditions (for example, cardiac arrest, mental change of 8 points or less in Glasgow coma scale, and shock status) among patients, with a critical first look. In most patients who are not in a very critical condition, the KTAS assessment starts with the main complaint of the patient; the KTAS then takes into account additional modifiers such as the vital signs, the level of consciousness, pain severity, injury mechanism, and blood sugar. Depending on which consideration is applied, the KTAS level can be evaluated diversely even in the same patient. In such a case, it is recommended that the more acute KTAS level be selected. Where pain severity is used as a modifier, the acuity level is determined depending on the location of pain, chronicity, and pain score. For example, in patients presenting with abdominal pain, if the onset of pain is acute and the pain score is moderate, the KTAS level is rated as 3. However, the degree of pain is a subjective factor that can be influenced by the patient's age, sex, race, psychological condition, and accompanying symptoms.[16–19] To minimize these problem, KTAS evaluators are trained through KTAS education program to use a 10-point Likert scale and a 10-cm visual analogue scale (VAS) when assessing the degree of the patient’s pain and to determine the final grade considering objective observations such as tachycardia and facial distortion, which better reflect the degree of pain rather than patient’s subjective experience. [9]

Despite these concerns, there is no research on the accuracy of triage when pain severity is used as the modifier in determining the final level of KTAS. The purpose of this study was to evaluate the accuracy of KTAS in predicting patient's severity when the degree of pain was used as a modifier.

## Materials and methods

### 1. Study design and setting

**Fig 1:**
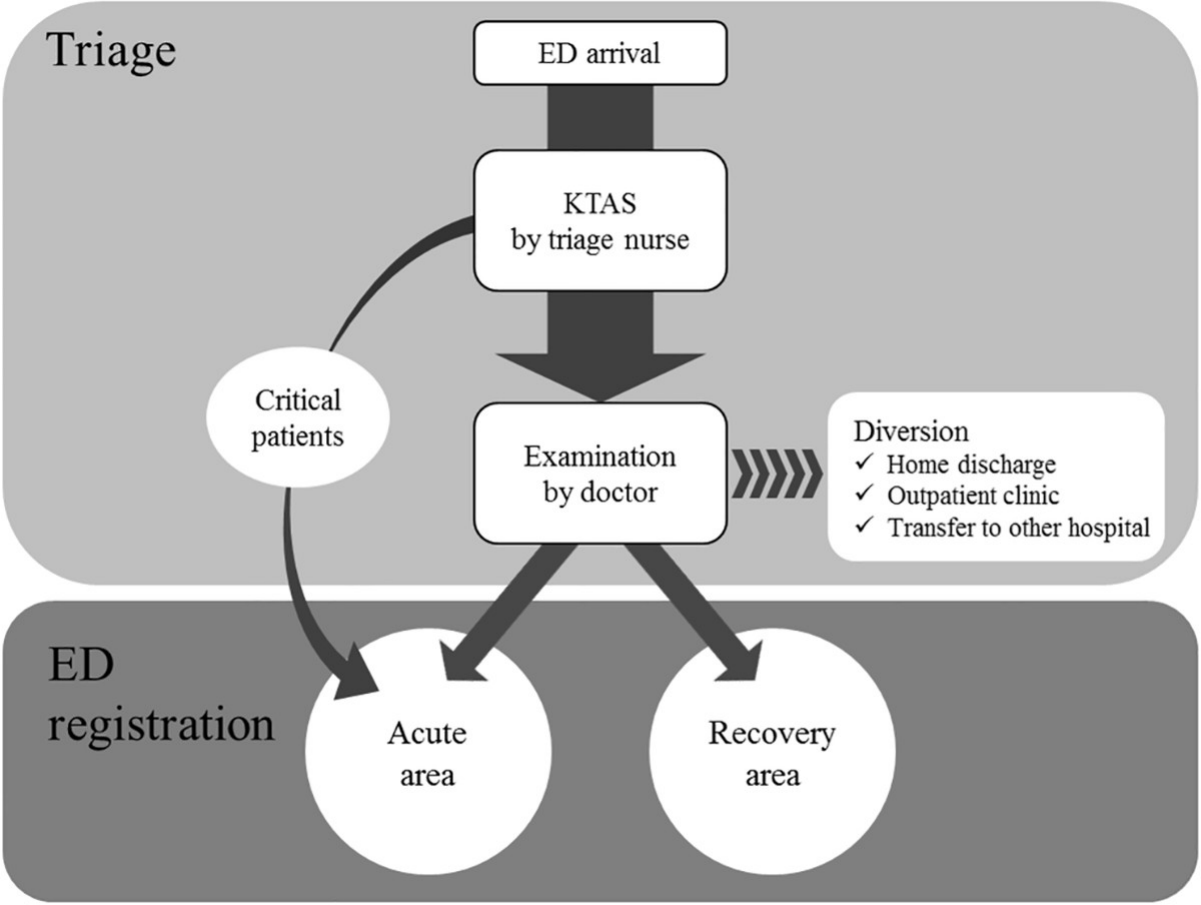
Triage and registration process in the emergency department.

This was a retrospective observational cohort study conducted in the ED of an urban tertiary university hospital with 2,000 beds, with more than 90,000 patients visiting the ED yearly; the hospital had separate adult and pediatric EDs, and patients aged over 16 years were treated in the adult ED. The adult ED was divided into an acute (with 17 beds) and recovery (with 26 beds and 10 clinic chairs) areas. We studied patients who were treated in the adult ED from January to June 2016. All data was collected anonymously, and this study was exempt from the obligation to obtain informed consent by the hospital’s institutional review board committee. When an emergency patient arrives at the ED, the triage nurse classifies the patient's severity using KTAS 1–5 (1 = resuscitation, 2 = emergency, 3 = urgent, 4 = less-urgent, 5 = non-urgent). Triage nurses were staff with more than four years of experience in the ED, and who had completed the 6-hour KTAS training program run by the KTAS committee under the Korean Emergency Medicine Association. Patients arriving with very urgent conditions such as unconsciousness and vital sign instability enter the acute area of the adult ED directly from the triage room (Fig 1). On the other hand, stable patients are sent to the doctor’s office and after examination by emergency physicians, they are then sent either to the acute or recovery area. Sometimes, the emergency physician may decide that the patient be diverted to the outpatient clinic or another hospital without ED registration, depending on the patient's condition or ED overcrowding.

We divided the patients into the pain and non-pain groups depending on whether the degree of pain was used or not used as a modifier in the KTAS evaluation. Patients who did not complain of pain and those whose consciousness was not clear were excluded because the evaluation of pain may not be reliable.

This study was approved by the hospital’s institutional review board (IRB) (Yonsei University Health System, Severance Hospital; Approval number: 4-2016-0987). All data were collected anonymously. This study was exempt from obtaining informed consent by the IRB committee.

### 2. Study variables

We extracted basic patient characteristics and medical information from the hospital information system. Non-medical problems mean that the patient’s disease was caused by external factors such as trauma, poisoning, and environmental factors. Complaint category referred to the body system that corresponds to the symptoms that the patient complains. The time from arrival to area registration was investigated in terms of the time required for triage and initial assessment. ED length of stay (LOS) was the time from ED arrival to hospitalization or discharge.

The lack of a complete standard reference for determining urgency has always been a problem in validation studies for triage tool.[13, 20] A criterion validity using expert opinion as reference standard and a construct validity using cost, resource use, hospitalization, and prognosis are available.[20] Since the triage system is used to rank the speed of care for the patient, that is, urgency, criterion validity is superior to construct validity. However, criterion validity study should be conducted proactively, and it also has the problem of inconsistency in expert opinions. We used emergency procedure, emergency operation, hospitalization, intensive care unit (ICU) admission, and 7-day mortality as indicators, which were the most commonly used in previous construct validity studies.[20] The emergency procedures include 43 life-saving procedures (such as cardiopulmonary resuscitation, endotracheal intubation, tracheotomy, defibrillation, central venous catheter insertion, pericardial puncture, and transcutaneous cardiac pacing) designated by the Korean government for receiving additional emergency fees. Emergency operation was limited to patients who were transferred directly from the ED to the operating room. Mortality was limited to within 7 days to reflect the death associated with acuity in the ED. We added one more indicator of acute area registration to better reflect the urgency of patients at the time of arrival at ED. For continuous vital sign monitoring was possible in the acute area, more urgent and more serious patients were assigned to the acute area according to the judgment of triage nurse or emergency physician at the time of triage or initial assessment. Finally, composite index was defined as all cases that corresponded to one or more of the above mentioned severity indicators.

### 3. Data analysis

Comparisons between the two pain groups were performed using chi-square analysis for categorical variables and Mann–Whitney U test for continuous variables because of the positive skewness in the data distribution. The distribution of KTAS and the proportion of urgent patients were compared between the two groups. Some differences in patients’ characteristics in both groups can affect urgency, and we have adjusted these characteristics. Potential factors were identified by using the Akaike information criterion (AIC) stepwise selection method and the adjusted odds ratio was presented through multivariable logistic regression. To evaluate the discriminative ability of KTAS, the adjusted odds ratios (OR) of each KTAS level compared to KTAS 3 for urgent patients were calculated. Since the number of patients with KTAS 3 was the greatest, KTAS 3 was used as a reference.[11] Influencing variables were selected using AIC, and the receiver operating characteristic (ROC) curves were generated to compare the predictive power of KTAS between the two groups. The areas under the ROC curves of each group were compared by the DeLong’s method. Statistical analyses were conducted using SAS (version 9.4, SAS Inc, Cary, NC, USA).

## Results

**Fig 2:**
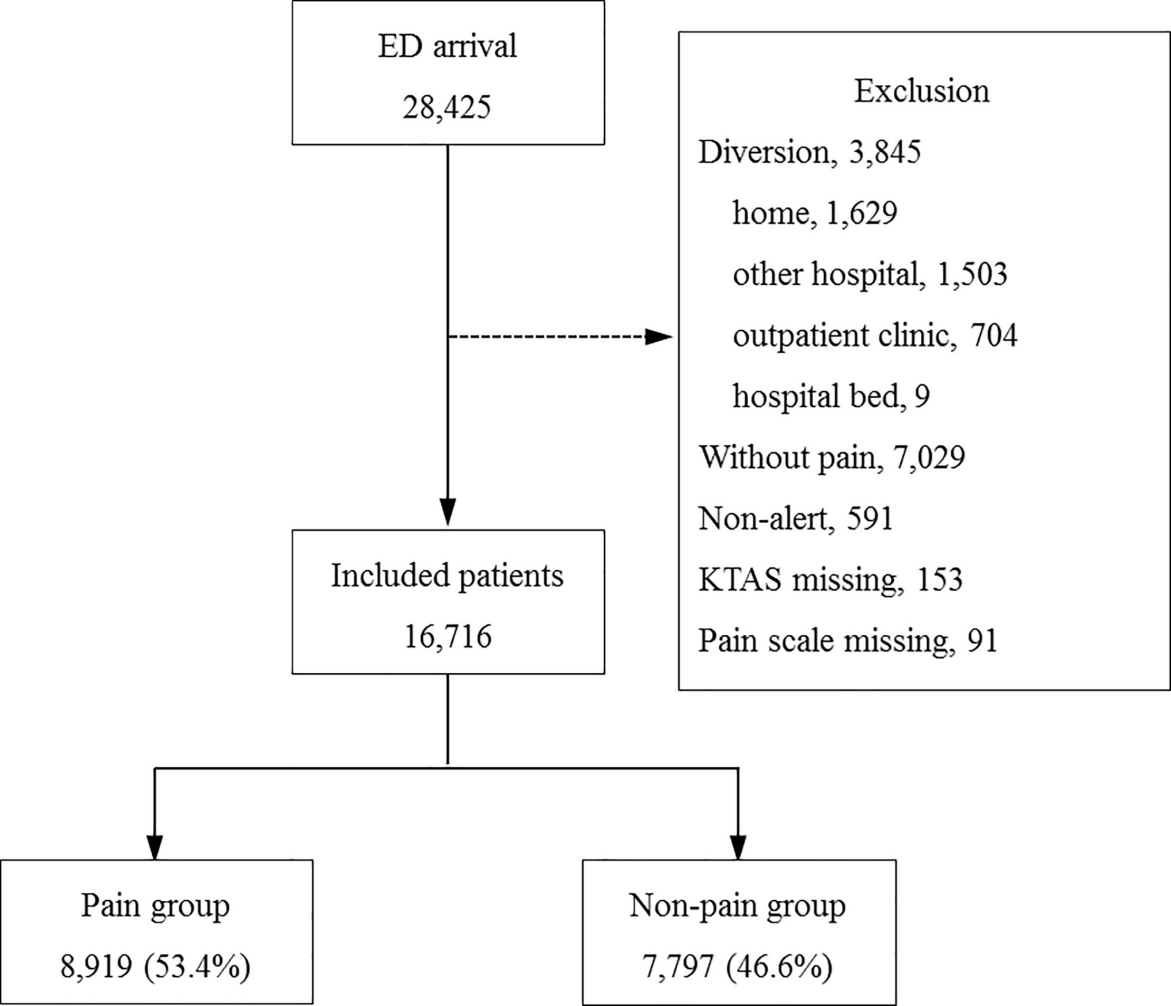
Number of patients included.

From January to June 2016, a total of 28,425 adult patients visited the ED (Fig 2). Among them, 3,845 (13.5%) patients were diverted; 1,629 (5.7%) patients who did not need emergency care were sent home, 1,503 (5.3%) were transferred to other hospitals, 704 (2.5%) were referred to the outpatient clinic, and 9 were admitted to the hospital bed directly. In 24,580 registered patients, 7,029 patients who did not complain of pain and 591 patients who had unconsciousness were excluded. We also excluded 153 patients with missing KTAS values and 91 patients without pain evaluation. Thus, 16,716 patients were included in the study; 8,919 (53.4%) in the pain group, and 7,797 (46.6%) in the non-pain group.

### 1. Patient characteristics

We compared the patient characteristics, KTAS data, and treatment outcomes between the two groups (Table 1). Patients in the pain group had more females and were younger than the non-pain group. In the pain group, fewer patients used ambulances (16.3% vs. 31.4%, p<0.001) and more patients had non-medical problems (24.6% vs. 18.6%, p<0.001). In the pain group, there were many cases of gastrointestinal and musculoskeletal symptoms, and in the non-pain group, many patients had general or cardiovascular symptoms. When the pain severity was used as a modifier, the KTAS was distributed only from 2 to 5 levels; hence, no patient was classified into KTAS 1 in the pain group. More patients were rated as more acute (KTAS 1–3) in the non-pain group (62.3% vs. 75.6%, p<0.001). Patients in the non-pain group had shorter time from arrival to ED registration (16 min vs. 13 min, p<0.001), but had longer LOS in the ED (222 min vs. 284 min, p<0.001).

**Table 1:** Comparison of patient characteristics between the two groups.

	Total N = 16,716	Pain N = 8,919	Non-Pain N = 7,797	P-value
Female, n (%)	8,877 (53.1)	4,912 (55.1)	3,965 (50.9)	<0.001
Age, median (IQR)	50 (32, 66)	45 (29, 63)	55 (35, 69)	<0.001
Ambulance arrival, n (%)	3,903 (23.3)	1,452 (16.3)	2,451 (31.4)	<0.001
Non-medical problem	3,642 (21.8)	2,195 (24.6)	1,447 (18.6)	<0.001
Complaint category				<0.001
Gastrointestinal	4,258 (25.5)	3,224 (36.1)	1,034 (13.3)	
Neurological	2,113 (12.6)	1,152 (12.9)	961 (12.3)	
General*	2,095 (12.5)	500 (5.6)	1,595 (20.5)	
Cardiovascular	1,553 (9.3)	405 (4.5)	1,148 (14.7)	
Musculoskeletal	2,092 (12.5)	1,598 (17.9)	494 (6.3)	
Respiratory	984 (5.9)	1 (0.0)	983 (12.6)	
Skin	1,064 (6.4)	290 (3.3)	774 (9.9)	
Others**	2,557 (15.3)	1,749 (19.6)	808 (10.4)	
KTAS level, n (%)				<0.001
1	167 (1.0)		167 (2.1)	
2	2,509 (15.0)	903 (10.1)	1,606 (20.6)	
3	8,776 (52.5)	4,655 (52.2)	4,121 (52.9)	
4	4,217 (25.2)	2,804 (31.4)	1,413 (18.1)	
5	1,047 (6.3)	557 (6.2)	490 (6.3)	
Arrival to registration (min), median (IQR)	15 (8, 37)	16 (9, 42)	13 (8, 30)	<0.001
ED LOS (min), median (IQR)	249 (141, 462)	222 (130, 403)	284 (160, 538)	<0.001
Severity variables				
Acute area registration, n (%)	3,475 (20.8)	818 (9.2)	2,657 (34.1)	<0.001
Emergency procedure, n (%)	1,026 (6.1)	275 (3.1)	751 (9.6)	<0.001
Emergency operation, n (%)	359 (2.1)	244 (2.7)	115 (1.5)	<0.001
Hospitalization, n (%)	4,791 (28.7)	2,036 (22.8)	2,755 (35.3)	<0.001
ICU admission, n (%)	599 (3.6)	145 (1.6)	454 (5.8)	<0.001
7-day mortality, n (%)	109 (0.7)	20 (0.2)	89 (1.1)	<0.001
Composite index, n (%)	6,334 (61.0)	2,479 (27.8)	3,855 (49.4)	<0.001

KTAS, Korean triage and acuity scale; ED, emergency department; LOS, length of stay; ICU, intensive care unit

*, not limited to a specific system

**, other categories not listed

Only 9.2% patients in the pain group were treated in the acute area, compared with 34.1% in the non-pain group (p<0.001). The number of patients who received emergency procedures in the ED was also higher in the non-pain group (3.1% vs. 9.6%, p<0.001). However, emergency operations were performed more in the pain group (2.7% vs. 1.5%, p<0.001). Patients in the non-pain group had higher rates of hospitalization, ICU admissions, hospital 7-day mortality, and composite index (27.8% vs. 49.4%, p<0.001).

### 2. Distribution of urgent patients in each KTAS level

The proportions of urgent patients in each KTAS level were compared between the two pain groups and shown in Table 2. In category KTAS 2, there were more urgent patients in the non-pain group with statistical significance for several indicators; acute area registration, emergency procedure, and hospitalization. However, emergency operation was performed in more patients in the pain group (3.7% vs. 2.2%, p<0.001). In the composite index, 41.9% urgent patients occurred in the pain group, while 81.4% urgent patients were in the non-pain group (p<0.001). In KTAS 3, the proportion of urgent patients was higher in the non-pain group for acute area registration, emergency procedure, hospitalization, and composite index. In patients rated as less-urgent (KTAS 4), more urgent patients occurred in the pain group for emergency operation(1.7% vs. 0.9%. p = 0.045). For other indicators, no significant differences were observed in KTAS 4. In KTAS 5, there was no significant difference between the two groups in all indicators. Statistical values of the variables included in multivariable logistic regression are given in S1–S7 Appendices.

**Table 2:** Comparison of severe patients within each KTAS between the two groups.

	Pain	Non-Pain	Adjusted OR P/NP (95% CI)	P-value
Acute area registration, n(%)				
KTAS 1		165/167 (98.8)		
2	179/903 (19.8)	1,185/1,606 (73.8)	0.31 (0.23–0.42)	<0.001
3	444/4,655 (9.5)	1,144/4,121 (27.8)	0.39 (0.33–0.45)	<0.001
4	175/2,804 (6.2)	135/1,413 (9.6)	0.83 (0.61–1.14)	0.249
5	20/557 (3.6)	28/490 (5.7)	0.62 (0.33–1.17)	0.141
Emergency procedure, n(%)				
KTAS 1		93/167 (55.7)		
2	54/903 (6.0)	293/1,606 (18.2)	0.35 (0.23–0.54)	<0.001
3	143/4,655 (3.1)	310/4,121 (7.5)	0.50 (0.39–0.64)	<0.001
4	69/2,804 (2.5)	49/1,413 (3.5)	0.78 (0.49–1.25)	0.297
5	9/557 (1.6)	6/490 (1.2)	1.47 (0.50–4.33)	0.483
Emergency operation, n(%)				
KTAS 1		4/167 (2.4)		
2	33/903 (3.7)	35/1,606 (2.2)	2.70 (1.56–4.67)	<0.001
3	159/4,655 (3.4)	61/4,121 (1.5)	2.73 (2.01–3.70)	<0.001
4	48/2,804 (1.7)	13/1,413 (0.9)	1.88 (1.01–3.48)	0.045
5	4/557 (0.7)	2/490 (0.4)	1.57 (0.28–8.69)	0.605
Hospitalization, n(%)				
KTAS 1		126/167 (75.5)		
2	307/903 (34.0)	847/1,606 (52.7)	0.68 (0.53–0.88)	0.003
3	1,271/4,655 (27.3)	1,537/4,121 (37.3)	0.74 (0.66–0.83)	<0.001
4	416/2,804 (14.8)	199/1,413 (14.1)	1.19 (0.93–1.53)	0.163
5	42/557 (7.5)	46/490 (9.4)	0.94 (0.59–1.52)	0.812
ICU admission, n(%)				
KTAS 1		45/167 (27.0)		
2	51/903 (5.7)	283/1,606 (17.6)	1.06 (0.71–1.56)	0.782
3	79/4,655 (1.7)	117/4,121 (2.8)	0.97 (0.69–1.36)	0.847
4	14/2,804 (0.5)	9/1,413 (0.6)	1.62 (0.51–5.11)	0.413
5	1/557 (0.2)	0/490 (0)		
7-day mortality, n(%)				
KTAS 1		21/167 (12.6)		
2	8/903 (0.9)	41/1,606 (2.6)	0.68 (0.30–1.56)	0.360
3	10/4,655 (0.2)	25/4,121 (0.6)	0.60 (0.28–1.27)	0.179
4	2/2,804 (0.1)	1/1,413 (0.1)	1.01 (0.09–11.12)	0.992
5	0/557 (0)	1/490 (0.2)		
Composite index, n(%)				
KTAS 1		100/167 (59.9)		
2	378/903 (41.9)	1,307/1,606 (81.4)	0.48 (0.36–0.64)	<0.001
3	1,498/4,655 (32.2)	2,013/4,121 (48.9)	0.63 (0.57–0.71)	<0.001
4	545/2,804 (19.4)	300/1,413 (21.2)	0.98 (0.79–1.21)	0.825
5	58/557 (10.4)	68/490 (13.9)	0.81 (0.54–1.23)	0.326

P, pain group; NP, non-pain group; OR, odds ratio; KTAS, Korean triage and acuity scale; ICU, intensive care unit

### 3. Predictability of KTAS for urgent patients

**Fig 3:**
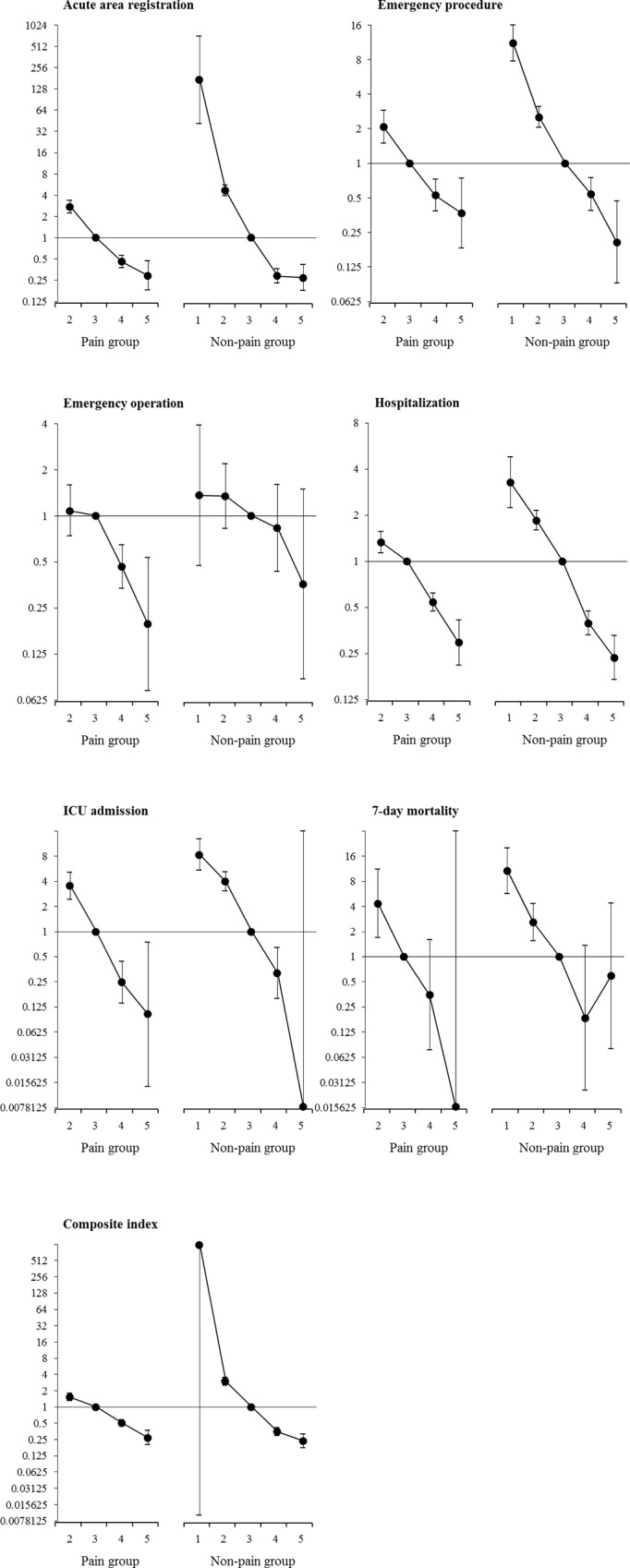
Adjusted odds ratios for urgent patients by Korean Triage and Acuity Scale (KTAS). Each plot represents the odds ratio and 95% confidence interval compared with KTAS 3. All 490 patients with KTAS 5 in the non-pain group did not admit to ICU, all 557 patients with KTAS 5 in the pain group did not expire within 7-day, and all 167 patients with KTAS 1 in the non-pain group were severe for the composite index. Therefore, the statistics were not calculated.

Correlation between KTAS and urgent patients are shown in Fig 3. The odds ratio of the occurrence of urgent patients decreased as KTAS levels increased in both groups. The difference between the odds ratios of each KTAS level was more evident in the non-pain group in acute area registration. emergency procedure, hospitalization, and composite index. The difference in severity of several indices between KTAS 2 and KTAS 3 was more clear in the non-pain group than in the pain group; adjusted OR (95% confidence interval (CI)) 2.73 (2.21–3.37) and 4.64 (3.92–5.51) for acute area registration, 2.07 (1.49–2.89) and 2.52 (2.05–3.09) for emergency procedure, 1.33 (1.13–1.56) and 1.85 (1.59–2.15) for hospitalization, 1.52 (1.30–1.78) and 2.98 (2.51–3.55) for composite index. The difference in severity between KTAS 3 and 4 was also greater in the non-pain group than in the pain group for several indicators; adjusted OR (95% CI) 0.46 (0.38–0.56) and 0.29 (0.23–0.36) for acute area registration, 0.54 (0.47–0.62) and 0.39 (0.33–0.47) for hospitalization, 0.51 (0.44–0.58) and 0.35 (0.30–0.42) for composite index.

**Fig 4:**
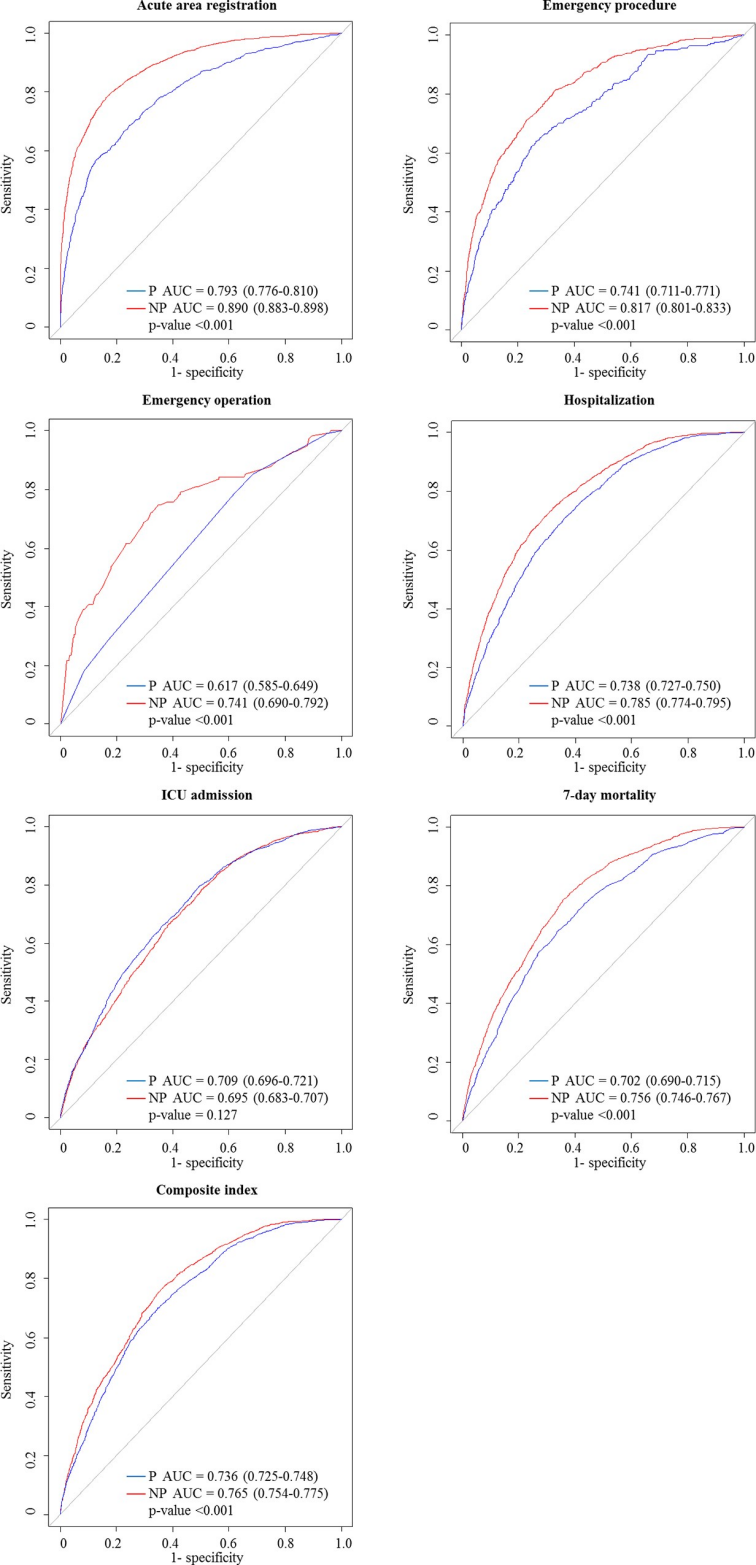
Comparison of receiver operating characteristic curve of Korean Triage and Acuity Scale predictability for patient urgency between the two groups.

Fig 4 shows the results of the AUC comparison of the predictive power of KTAS for urgent patients between the two groups. KTAS showed good level of predictive power with AUC 0.8 or higher for acute area registration and emergency procedure in non-pain group. The predictability of KTAS for urgent patients in the non-pain group was higher than that in the pain group in acute area registration, emergency procedure, emergency operation, hospitalization, 7-day mortality, and composite index with statistical significance. Statistical values of the variables included in multivariable logistic regression of Figs 3 and 4 are listed in S8–S14 Appendices.

## Discussion

When the severity was classified using KTAS in emergency patients with pain, pain severity was used as a modifier in 53.4% of patients and their severity was lower than that of patients who were evaluated regardless of pain severity. The predictive power of KTAS in urgent patients was higher in the non-pain group for several severity indicators. We found that the acuity level was overestimated in the pain group patients. The ideal triage tool should be able to constantly evaluate the urgency of the patient; hence, the same triage level should indicate the same urgency. In particular, when the ED is overcrowded, it is critical to ensure that the limited resources are preferentially provided to more urgent patients; this underscores the importance of triage as a starting point in determining severity. Several factors may be related to the accuracy of triage. We have confirmed that pain severity as a modifier in KTAS is a factor affecting accuracy. The cause of over-triage by applying the degree of pain to KTAS evaluation can be considered diversely.

At the time of arrival at the ED, patients are often anxious, due to the unfamiliar ED environment and the uncertainty of their health conditions, and this anxiety can worsen their pain.[21, 22] As reported, in particular, women and young people are more susceptible to pain due to various physiological and psychosocial reasons; the pain group in this study included more of such patients.[17, 19] It is also difficult for patients to objectively rate their own pain score due to inaccurate medical knowledge about pain scores. Since it is important to consult with patients on their level of pain, it is also important to determine how the patients are asked about the level of pain. However, it is difficult to educate patients adequately in a situation where triage need to be performed within a short time.[23] On the other hand, patients may intentionally exaggerate their symptoms at triage in order to obtain medical treatment more quickly. Sometimes patients with mild pain may exaggerate their symptoms to justify their use of the emergency room.[24] According to previous studies, triage nurses and doctors rated the pain intensity lower than the patient's experience.[23, 25] Even in a study on the MTS, pain assessment at triage was not performed in about 68% of patients, with concern that pain assessment could lead to over-triage.[26] Especially in Korea, where the non-emergency patients with KTAS 4 and 5 have lower national health insurance coverage rates, and patient's medical expenses increase by about twice as much, patients might therefore want to be seen as more urgent patients with a more acute KTAS score.

To prevent over-triage in patients with pain, there is a need to more aggressively apply objective indicators such as facial distortion and vital signs to calibrate the patient's pain level. However, as reported by Guru et al., pain scores for the same patient differ between medical staffs.[25] For other modifiers of KTAS other than pain, class-specific examples are provided in the guideline such that relatively consistent assessments can be made.[9] For example, in the case of breathing, severe dyspnea means that the patient is unable to speak or can speak only one word. Moderate dyspnea is a condition in which the patient can only make a short sentence or a conversation that can be interrupted, while mild dyspnea means frequent breathing, although, conversation is possible.[9] However, with pain, no standard exists on how an objective assessment could be applied to adjust the pain score reported by patients. Therefore, it is necessary to establish a clinical definition for the patient's appearance and physiological response to each severe, moderate, or mild pain, such that the medical staffs can make consistent assessments.

It is also necessary to evaluate whether the 4 and 8-point boundary distinguishing between mild, moderate, and severe pain in KTAS is appropriate. In ATS and MTS (similar to KTAS), the triage levels of patients with pain is determined according to the three stages of severe, moderate, and mild pain. However, the boundaries for dividing the degree of pain differs; compared to KTAS, ATS applies the lower scores of 4 and 7-point while MTS has the higher scores of 5 and 8-point.[27] KTAS applied pain related items of CTAS in the same way without any modification in the boundaries of pain severity, how to measure pain, and evaluator training. The pain threshold of KTAS may need to be adjusted, as racial differences can affect the perception of pain intensity.[28, 29] In ESI, the influence of the pain score on the severity classification is relatively low compared to other triage tools, because the pain scores are applied in a different way; while ESI 2 is recommended for patients with a pain score of 7 or greater, pain severity is not considered when classifying ESI 3–5.[30] One study reported that there was no correlation between the pain score and ESI triage categories.[31] The degree of pain, regardless of the severity in the patient or the need for admission, may reflect urgency in the sense that pain should be reduced quickly.[32] However, if the reliability of triage is compromised by the patient's pain, the value of triage as an effective communication tool for prioritizing emergency care might be reduced. Therefore, when applying the degree of pain to the KTAS classification, it is necessary to objectively measure the degree of pain and to calibrate the triage level so as not to overestimate it. This study has several limitations. First, since this study was conducted in a large university hospital with a lot of seriously ill patients and a high degree of ED overcrowding, it cannot be regarded as representing the ED in Korea. Second, we did not address the triage nurse’s ability to assess patients' pain. The results may vary depending on how the triage nurse communicated with the patient in order to accurately express the patients’ own pain level, or how aggressively the exaggerated pain level could be corrected. Third, indicators such as hospitalization or 7-day mortality which signifies the outcome of treatment may not accurately reflect the urgency of the patient. The acute area registration used as an indicator in this study can be seen as a judgment of the emergency medical staff who treated the patient at that time. However, there is a limitation that area allocation in the ED can be affected by the medical staff experience and the degree of ED overcrowding at the time. Fourth, some differences were found in various basic demographic information of two comparative groups. Although statistical analysis was performed by adjusting for these differences, there is still some limitation in that other differences that may exist between the two groups could not be corrected due to the retrospective nature of this study. Finally, the specific situation in Korea, where the KTAS rating affects the patient's medical expenditure, may have affected the outcomes by exaggerating the patient's pain level.

### Conclusions

The severity of patient was overestimated and the accuracy of KTAS for predicting urgent patients was reduced when the patient's pain level was used as a modifier in the severity classification. In order to adequately consider the patient's pain level to the severity classification, the degree of pain should be accurately assessed and, if necessary, KTAS guideline should be supplemented, to correct the overestimation. A future research using a prospective observational study would be necessary to estimate the ability of KTAS in predicting urgent patients with patient’s level of pain as a modifier more accurately.

## Supporting information

S1 Appendix Analysis result by multivariable logistic regression for acute area registration for Table 2. KTAS, Korean triage and acuity scale; OR, odds ratio; CI, confidence interval; The reference value for complaint category is Gastrointestinal.
(DOCX)

S2 Appendix Analysis result by multivariable logistic regression for emergency procedure for Table 2. KTAS, Korean triage and acuity scale; OR, odds ratio; CI, confidence interval; The reference value for complaint category is Gastrointestinal.
(DOCX)

S3 Appendix Analysis result by multivariable logistic regression for emergency operation for Table 2. KTAS, Korean triage and acuity scale; OR, odds ratio; CI, confidence interval; The reference value for complaint category is Gastrointestinal.
(DOCX)

S4 Appendix Analysis result by multivariable logistic regression for hospitalization for Table 2. KTAS, Korean triage and acuity scale; OR, odds ratio; CI, confidence interval; The reference value for complaint category is Gastrointestinal.
(DOCX)

S5 Appendix Analysis result by multivariable logistic regression for ICU admission for Table 2. ICU, intensive care unit; KTAS, Korean triage and acuity scale; OR, odds ratio; CI, confidence interval; The reference value for complaint category is Gastrointestinal.
(DOCX)

S6 Appendix Analysis result by multivariable logistic regression for 7-day mortality for Table 2. KTAS, Korean triage and acuity scale; OR, odds ratio; CI, confidence interval; The reference value for complaint category is Gastrointestinal.
(DOCX)

S7 Appendix Analysis result by multivariable logistic regression for composite index for Table 2. KTAS, Korean triage and acuity scale; OR, odds ratio; CI, confidence interval; The reference value for complaint category is Gastrointestinal.
(DOCX)

S8 Appendix Analysis result by multivariable logistic regression for acute area registration for Figs 3 and 4. KTAS, Korean triage and acuity scale; OR, odds ratio; CI, confidence interval; The reference value for complaint category is Gastrointestinal.
(DOCX)

S9 Appendix Analysis result by multivariable logistic regression for emergency procedure for Figs 3 and 4. KTAS, Korean triage and acuity scale; OR, odds ratio; CI, confidence interval; The reference value for complaint category is Gastrointestinal.
(DOCX)

S10 Appendix Analysis result by multivariable logistic regression for emergency operation for Figs 3 and 4. KTAS, Korean triage and acuity scale; OR, odds ratio; CI, confidence interval; The reference value for complaint category is Gastrointestinal.
(DOCX)

S11 Appendix Analysis result by multivariable logistic regression for hospitalization for Figs 3 and 4. KTAS, Korean triage and acuity scale; OR, odds ratio; CI, confidence interval; The reference value for complaint category is Gastrointestinal.
(DOCX)

S12 Appendix Analysis result by multivariable logistic regression for ICU admission for Figs 3 and 4. ICU, intensive care unit; KTAS, Korean triage and acuity scale; OR, odds ratio; CI, confidence interval; The reference value for complaint category is Gastrointestinal.; All 490 patients with KTAS 5 in the non-pain group did not admit to ICU, so the statistics were not calculated.
(DOCX)

S13 Appendix Analysis result by multivariable logistic regression for 7-day mortality for Figs 3 and 4. KTAS, Korean triage and acuity scale; OR, odds ratio; CI, confidence interval; The reference value for complaint category is Gastrointestinal. All 557 patients with KTAS 5 in the pain group did not expire within 7-day, so the statistics were not calculated.
(DOCX)

S14 Appendix Analysis result by multivariable logistic regression for composite index for Figs 3 and 4. KTAS, Korean triage and acuity scale; OR, odds ratio; CI, confidence interval; The reference value for complaint category is Gastrointestinal.; All 167 patients in the non-pain group with KTAS 1 were severe, so the statistics were not calculated.
(DOCX)
