# Supplementary material for: Over-triage occurs when considering the patient's pain in Korean Triage and Acuity Scale (KTAS)
Source: PLoS One. 2019 May 9;14(5):e0216519. doi: 10.1371/journal.pone.0216519 (PMC6508716; doi:10.1371/journal.pone.0216519)
Supplement: S1 Appendix — KTAS, Korean triage and acuity scale; OR, odds ratio; CI, confidence interval; The reference value for complaint category is Gastrointestinal. (DOCX) [file pone.0216519.s001.docx]

| KTAS | Variable | OR (95% CI) | p-value |
| --- | --- | --- | --- |
| KTAS 2 | Pain group | 0.31 (0.23-0.42) | <0.001 |
|  | Non-medical problem | 2.45 (1.54-3.89) | <0.001 |
|  | Complaint (Respiratory) | 13.20 (5.15-33.87) | <0.001 |
|  | Complaint (Cardiovascular) | 4.87 (3.27-7.25) | <0.001 |
|  | Complaint (Neurological) | 1.01 (0.65-1.55) | 0.975 |
|  | Complaint (Musculoskeletal) | 0.19 (0.08-0.43) | <0.001 |
|  | Complaint (Skin) | 0.64 (0.24-1.74) | 0.384 |
|  | Complaint (General) | 0.75 (0.45-1.24) | 0.257 |
|  | Complaint (Others) | 0.40 (0.24-0.67) | <0.001 |
|  | Female | 0.77 (0.61-0.98) | 0.033 |
|  | Age | 1.03 (1.02-1.04) | <0.001 |
|  | Ambulance arrival | 18.04 (13.46-24.19) | <0.001 |
| KTAS 3 | Pain group | 0.39 (0.33-0.45) | <0.001 |
|  | Non-medical problem | 1.21 (0.95-1.53) | 0.126 |
|  | Complaint (Respiratory) | 1.83 (1.45-2.31) | <0.001 |
|  | Complaint (Cardiovascular) | 3.65 (2.83-4.70) | <0.001 |
|  | Complaint (Neurological) | 1.01 (0.83-1.22) | 0.963 |
|  | Complaint (Musculoskeletal) | 0.34 (0.24-0.47) | <0.001 |
|  | Complaint (Skin) | 0.62 (0.36-1.09) | 0.098 |
|  | Complaint (General) | 0.88 (0.70-1.10) | 0.255 |
|  | Complaint (Others) | 0.47 (0.36-0.61) | <0.001 |
|  | Female | 0.71 (0.62-0.81) | <0.001 |
|  | Age | 1.02 (1.02-1.03) | <0.001 |
|  | Ambulance arrival | 7.90 (6.90-9.05) | <0.001 |
| KTAS 4 | Pain group | 0.83 (0.61-1.14) | 0.249 |
|  | Complaint (Respiratory) | 4.70 (2.52-8.76) | <0.001 |
|  | Complaint (Cardiovascular) | 5.37 (3.27-8.81) | <0.001 |
|  | Complaint (Neurological) | 1.75 (1.03-2.99) | 0.040 |
|  | Complaint (Musculoskeletal) | 0.98 (0.63-1.53) | 0.934 |
|  | Complaint (Skin) | 0.53 (0.28-1.03) | 0.060 |
|  | Complaint (General) | 1.30 (0.78-2.16) | 0.316 |
|  | Complaint (Others) | 1.12 (0.70-1.81) | 0.634 |
|  | Female | 0.81 (0.63-1.04) | 0.099 |
|  | Age | 1.02 (1.02-1.03) | <0.001 |
|  | Ambulance arrival | 4.46 (3.42-5.81) | <0.001 |
| KTAS 5 | Pain group | 0.62 (0.33-1.17) | 0.141 |
|  | Non-medical problem | 0.46 (0.23-0.93) | 0.031 |
|  | Ambulance arrival | 6.56 (3.59-12.00) | <0.001 |
