# Supplementary material for: Over-triage occurs when considering the patient's pain in Korean Triage and Acuity Scale (KTAS)
Source: PLoS One. 2019 May 9;14(5):e0216519. doi: 10.1371/journal.pone.0216519 (PMC6508716; doi:10.1371/journal.pone.0216519)
Supplement: S2 Appendix — KTAS, Korean triage and acuity scale; OR, odds ratio; CI, confidence interval; The reference value for complaint category is Gastrointestinal. (DOCX) [file pone.0216519.s002.docx]

| KTAS | Variable | OR (95% CI) | p-value |
| --- | --- | --- | --- |
| KTAS 2 | Pain group | 0.35 (0.23-0.54) | <0.001 |
|  | Non-medical problem | 3.83 (2.32-6.32) | <0.001 |
|  | Complaint (Respiratory) | 0.48 (0.28-0.84) | 0.01 |
|  | Complaint (Cardiovascular) | 0.19 (0.12-0.31) | <0.001 |
|  | Complaint (Neurological) | 0.20 (0.11-0.35) | <0.001 |
|  | Complaint (Musculoskeletal) | 0.24 (0.10-0.58) | 0.002 |
|  | Complaint (Skin) | 0.11 (0.01-0.88) | 0.037 |
|  | Complaint (General) | 0.69 (0.41-1.17) | 0.167 |
|  | Complaint (Others) | 0.36 (0.20-0.66) | 0.001 |
|  | Female | 0.58 (0.45-0.75) | <0.001 |
|  | Age | 1.02 (1.01-1.03) | <0.001 |
|  | Ambulance arrival | 3.96 (2.95-5.31) | <0.001 |
| KTAS 3 | Pain group | 0.50 (0.39-0.64) | <0.001 |
|  | Non-medical problem | 2.87 (2.05-4.03) | <0.001 |
|  | Complaint (Respiratory) | 1.35 (0.98-1.88) | 0.069 |
|  | Complaint (Cardiovascular) | 1.22 (0.83-1.80) | 0.313 |
|  | Complaint (Neurological) | 0.47 (0.33-0.66) | <0.001 |
|  | Complaint (Musculoskeletal) | 0.25 (0.15-0.42) | <0.001 |
|  | Complaint (Skin) | 0.15 (0.05-0.51) | 0.002 |
|  | Complaint (General) | 0.77 (0.55-1.07) | 0.120 |
|  | Complaint (Others) | 0.36 (0.24-0.55) | <0.001 |
|  | Female | 0.54 (0.44-0.65) | <0.001 |
|  | Age | 1.01 (1.01-1.02) | <0.001 |
|  | Ambulance arrival | 2.80 (2.28-3.45) | <0.001 |
| KTAS 4 | Pain group | 0.78 (0.49-1.25) | 0.297 |
|  | Non-medical problem | 3.14 (1.91-5.17) | <0.001 |
|  | Complaint (Respiratory) | 2.75 (0.98-7.75) | 0.056 |
|  | Complaint (Cardiovascular) | 1.57 (0.63-3.91) | 0.336 |
|  | Complaint (Neurological) | 1.32 (0.56-3.12) | 0.530 |
|  | Complaint (Musculoskeletal) | 0.47 (0.21-1.06) | 0.070 |
|  | Complaint (Skin) | 0.19 (0.06-0.62) | 0.006 |
|  | Complaint (General) | 1.73 (0.79-3.75) | 0.168 |
|  | Complaint (Others) | 1.18 (0.55-2.53) | 0.665 |
|  | Female | 0.66 (0.45-0.97) | 0.036 |
|  | Age | 1.01(1.00-1.02) | 0.047 |
|  | Ambulance arrival | 5.80 (3.81-8.83) | <0.001 |
| KTAS 5 | Pain group | 1.47 (0.50-4.33) | 0.483 |
|  | Non-medical problem | 0.42 (0.13-1.38) | 0.153 |
|  | Ambulance arrival | 4.30 (1.53-12.12) | 0.006 |
