# Supplementary material for: Over-triage occurs when considering the patient's pain in Korean Triage and Acuity Scale (KTAS)
Source: PLoS One. 2019 May 9;14(5):e0216519. doi: 10.1371/journal.pone.0216519 (PMC6508716; doi:10.1371/journal.pone.0216519)
Supplement: S3 Appendix — KTAS, Korean triage and acuity scale; OR, odds ratio; CI, confidence interval; The reference value for complaint category is Gastrointestinal. (DOCX) [file pone.0216519.s003.docx]

| KTAS | Variable | OR (95% CI) | p-value |
| --- | --- | --- | --- |
| KTAS 2 | Pain group | 2.70 (1.56-4.67) | <0.001 |
|  | Non-medical problem | 4.35 (2.51-7.54) | <0.001 |
|  | Ambulance arrival | 2.14 (1.23-3.72) | 0.007 |
| KTAS 3 | Pain group | 2.73 (2.01-3.70) | <0.001 |
|  | Ambulance arrival | 2.45 (1.83-3.29) | <0.001 |
| KTAS4 | Pain group | 1.88(1.01-3.48) | 0.045 |
|  | Age | 1.02 (1.01-1.03) | 0.001 |
| KTAS5 | Pain group | 1.57 (0.28-8.69) | 0.605 |
|  | Ambulance arrival | 4.93 (0.98-24.79) | 0.053 |
