# Supplementary material for: Over-triage occurs when considering the patient's pain in Korean Triage and Acuity Scale (KTAS)
Source: PLoS One. 2019 May 9;14(5):e0216519. doi: 10.1371/journal.pone.0216519 (PMC6508716; doi:10.1371/journal.pone.0216519)
Supplement: S4 Appendix — KTAS, Korean triage and acuity scale; OR, odds ratio; CI, confidence interval; The reference value for complaint category is Gastrointestinal. (DOCX) [file pone.0216519.s004.docx]

| KTAS | Variable | OR (95% CI) | p-value |
| --- | --- | --- | --- |
| KTAS 2 | Pain group | 0.68 (0.53-0.88) | 0.003 |
|  | Complaint (Respiratory) | 1.42 (0.87-2.30) | 0.157 |
|  | Complaint (Cardiovascular) | 0.53 (0.39-0.72) | <0.001 |
|  | Complaint (Neurological) | 0.35 (0.25-0.49) | <0.001 |
|  | Complaint (Musculoskeletal) | 0.35 (0.20-0.60) | <0.001 |
|  | Complaint (Skin) | 0.09 (0.02-0.39) | 0.001 |
|  | Complaint (General) | 1.32 (0.89-1.96) | 0.161 |
|  | Complaint (Others) | 0.41 (0.3-0.57) | <0.001 |
|  | Female | 0.72 (0.61-0.86) | <0.001 |
|  | Age | 1.02 (1.02-1.03) | <0.001 |
|  | Ambulance arrival | 2.22 (1.83-2.69) | <0.001 |
| KTAS 3 | Pain group | 0.74 (0.66-0.83) | <0.001 |
|  | Non-medical problem | 0.37 (0.30-0.45) | <0.001 |
|  | Complaint (Respiratory) | 0.91 (0.75-1.12) | 0.376 |
|  | Complaint (Cardiovascular) | 0.61 (0.49-0.77) | <0.001 |
|  | Complaint (Neurological) | 0.35 (0.30-0.41) | <0.001 |
|  | Complaint (Musculoskeletal) | 0.64 (0.51-0.80) | <0.001 |
|  | Complaint (Skin) | 0.36 (0.22-0.61) | <0.001 |
|  | Complaint (General) | 0.89 (0.75-1.05) | 0.154 |
|  | Complaint (Others) | 0.50 (0.42-0.59) | <0.001 |
|  | Female | 0.67 (0.61-0.74) | <0.001 |
|  | Age | 1.02 (1.02-1.03) | <0.001 |
|  | Ambulance arrival | 2.29 (2.03-2.58) | <0.001 |
| KTAS 4 | Pain group | 1.19 (0.93-1.53) | 0.163 |
|  | Non-medical problem | 0.44 (0.34-0.58) | <0.001 |
|  | Complaint (Respiratory) | 1.69 (1.05-2.74) | 0.032 |
|  | Complaint (Cardiovascular) | 0.69 (0.46-1.02) | 0.063 |
|  | Complaint (Neurological) | 0.34 (0.21-0.55) | <0.001 |
|  | Complaint (Musculoskeletal) | 0.61 (0.45-0.82) | 0.001 |
|  | Complaint (Skin) | 0.15 (0.09-0.26) | <0.001 |
|  | Complaint (General) | 0.69 (0.50-0.94) | 0.019 |
|  | Complaint (Others) | 0.26 (0.18-0.37) | <0.001 |
|  | Female | 0.78 (0.65-0.94) | 0.009 |
|  | Age | 1.04 (1.03-1.04) | <0.001 |
|  | Ambulance arrival | 1.69 (1.37-2.08) | <0.001 |
| KTAS 5 | Pain group | 0.94 (0.59-1.52) | 0.812 |
|  | Non-medical problem | 0.52 (0.30-0.90) | 0.018 |
|  | Age | 1.04 (1.02-1.05) | <0.001 |
|  | Ambulance arrival | 2.60 (1.57-4.30) | <0.001 |
