# Supplementary material for: Over-triage occurs when considering the patient's pain in Korean Triage and Acuity Scale (KTAS)
Source: PLoS One. 2019 May 9;14(5):e0216519. doi: 10.1371/journal.pone.0216519 (PMC6508716; doi:10.1371/journal.pone.0216519)
Supplement: S5 Appendix — ICU, intensive care unit; KTAS, Korean triage and acuity scale; OR, odds ratio; CI, confidence interval; The reference value for complaint category is Gastrointestinal. (DOCX) [file pone.0216519.s005.docx]

| KTAS | Variable | OR (95% CI) | p-value |
| --- | --- | --- | --- |
| KTAS 2 | Pain group | 1.06 (0.71-1.56) | 0.782 |
|  | Non-medical problem | 2.40 (1.38-4.20) | 0.002 |
|  | Complaint (Respiratory) | 2.77 (1.4-5.47) | 0.003 |
|  | Complaint (Cardiovascular) | 4.63 (2.65-8.07) | <0.001 |
|  | Complaint (Neurological) | 2.95 (1.62-5.39) | <0.001 |
|  | Complaint (Musculoskeletal) | 0.49 (0.13-1.83) | 0.290 |
|  | Complaint (Skin) | 1.41 (0.17-11.77) | 0.750 |
|  | Complaint (General) | 0.88 (0.40-1.96) | 0.755 |
|  | Complaint (Others) | 1.27 (0.61-2.67) | 0.524 |
|  | Female | 0.47 (0.36-0.62) | <0.001 |
|  | Age | 1.02 (1.02-1.03) | <0.001 |
|  | Ambulance arrival | 3.50 (2.63-4.64) | <0.001 |
| KTAS 3 | Pain group | 0.97 (0.69-1.36) | 0.847 |
|  | Non-medical problem | 0.66 (0.39-1.13) | 0.131 |
|  | Complaint (Respiratory) | 1.89 (1.02-3.51) | 0.044 |
|  | Complaint (Cardiovascular) | 3.52 (1.93-6.44) | <0.001 |
|  | Complaint (Neurological) | 6.55 (4.30-9.98) | <0.001 |
|  | Complaint (Musculoskeletal) | 1.57 (0.73-3.39) | 0.252 |
|  | Complaint (Skin) | 0.95 (0.13-7.27) | 0.962 |
|  | Complaint (General) | 0.67 (0.30-1.50) | 0.335 |
|  | Complaint (Others) | 1.07 (0.53-2.16) | 0.847 |
|  | Female | 0.54 (0.40-0.72) | <0.001 |
|  | Age | 1.03 (1.02-1.04) | <0.001 |
|  | Ambulance arrival | 2.60 (1.91-3.54) | <0.001 |
| KTAS 4 | Pain group | 1.62 (0.51-5.11) | 0.413 |
|  | Complaint (Respiratory) | 10.84 (1.51-77.86) | 0.018 |
|  | Complaint (Cardiovascular) | 6.47 (1.12-37.31) | 0.037 |
|  | Complaint (Neurological) | 5.66 (1.00-32.09) | 0.05 |
|  | Complaint (Musculoskeletal) | 1.43 (0.27-7.59) | 0.674 |
|  | Complaint (Skin) | 1.01 (0.09-11.91) | 0.995 |
|  | Complaint (General) | 0.74 (0.07-8.37) | 0.804 |
|  | Complaint (Others) | 1.01 (0.14-7.32) | 0.992 |
|  | Female | 0.42 (0.17-1.01) | 0.052 |
|  | Age | 1.08 (1.04-1.11) | <0.001 |
| KTAS 5 | Pain group | - | - |
