# Supplementary material for: Over-triage occurs when considering the patient's pain in Korean Triage and Acuity Scale (KTAS)
Source: PLoS One. 2019 May 9;14(5):e0216519. doi: 10.1371/journal.pone.0216519 (PMC6508716; doi:10.1371/journal.pone.0216519)
Supplement: S6 Appendix — KTAS, Korean triage and acuity scale; OR, odds ratio; CI, confidence interval; The reference value for complaint category is Gastrointestinal. (DOCX) [file pone.0216519.s006.docx]

| KTAS | Variable | OR (95% CI) | p-value |
| --- | --- | --- | --- |
| KTAS 2 | Pain group | 0.68 (0.30-1.56) | 0.360 |
|  | Non-medical problem | 0.19 (0.03-1.38) | 0.100 |
|  | Age | 1.03 (1.01-1.05) | 0.010 |
|  | Ambulance arrival | 2.93 (1.47-5.83) | 0.002 |
| KTAS 3 | Pain group | 0.60 (0.28-1.27) | 0.179 |
|  | Non-medical problem | 0.21 (0.03-1.51) | 0.120 |
|  | Female | 0.52 (0.26-1.05) | 0.069 |
|  | Age | 1.04 (1.02-1.07) | <0.001 |
|  | Ambulance arrival | 4.14 (2.02-8.48) | <0.001 |
| KTAS 4 | Pain group | 1.01 (0.09-11.12) | 0.995 |
| KTAS 5 | Pain group | Unpredictable | 0.998 |
|  | Ambulance arrival | Unpredictable | 0.998 |
