# Supplementary material for: Over-triage occurs when considering the patient's pain in Korean Triage and Acuity Scale (KTAS)
Source: PLoS One. 2019 May 9;14(5):e0216519. doi: 10.1371/journal.pone.0216519 (PMC6508716; doi:10.1371/journal.pone.0216519)
Supplement: S7 Appendix — KTAS, Korean triage and acuity scale; OR, odds ratio; CI, confidence interval; The reference value for complaint category is Gastrointestinal. (DOCX) [file pone.0216519.s007.docx]

| KTAS | Variable | OR (95% CI) | p-value |
| --- | --- | --- | --- |
| KTAS 2 | Pain group | 0.48 (0.36-0.64) | <0.001 |
|  | Complaint (Respiratory) | 7.78 (2.31-26.22) | <0.001 |
|  | Complaint (Cardiovascular) | 1.77 (1.24-2.54) | 0.002 |
|  | Complaint (Neurological) | 0.44 (0.30-0.63) | <0.001 |
|  | Complaint (Musculoskeletal) | 0.40 (0.22-0.73) | 0.003 |
|  | Complaint (Skin) | 0.32 (0.13-0.78) | 0.012 |
|  | Complaint (General) | 1.12 (0.71-1.77) | 0.622 |
|  | Complaint (Others) | 0.39 (0.27-0.56) | <0.001 |
|  | Female | 0.73 (0.59-0.91) | 0.004 |
|  | Age | 1.02 (1.02-1.03) | <0.001 |
|  | Ambulance arrival | 12.82 (9.27-17.73) | <0.001 |
| KTAS 3 | Pain group | 0.63 (0.57-0.71) | <0.001 |
|  | Non-medical problem | 0.59 (0.49-0.70) | <0.001 |
|  | Complaint (Respiratory) | 1.21 (0.98-1.48) | 0.078 |
|  | Complaint (Cardiovascular) | 1.52 (1.22-1.90) | <0.001 |
|  | Complaint (Neurological) | 0.49 (0.42-0.56) | <0.001 |
|  | Complaint (Musculoskeletal) | 0.46 (0.37-0.58) | <0.001 |
|  | Complaint (Skin) | 0.43 (0.27-0.67) | <0.001 |
|  | Complaint (General) | 0.88 (0.74-1.04) | 0.120 |
|  | Complaint (Others) | 0.47 (0.4-0.56) | <0.001 |
|  | Female | 0.66 (0.6-0.73) | <0.001 |
|  | Age | 1.03 (1.02-1.03) | <0.001 |
|  | Ambulance arrival | 4.56 (4.01-5.17) | <0.001 |
| KTAS 4 | Pain group | 0.98 (0.79-1.21) | 0.825 |
|  | Non-medical problem | 0.65 (0.52-0.82) | <0.001 |
|  | Complaint (Respiratory) | 2.02 (1.26-3.23) | 0.003 |
|  | Complaint (Cardiovascular) | 1.65 (1.15-2.36) | 0.006 |
|  | Complaint (Neurological) | 0.60 (0.40-0.89) | 0.011 |
|  | Complaint (Musculoskeletal) | 0.61 (0.45-0.81) | <0.001 |
|  | Complaint (Skin) | 0.21 (0.14-0.33) | <0.001 |
|  | Complaint (General) | 0.75 (0.55-1.01) | 0.058 |
|  | Complaint (Others) | 0.41 (0.30-0.55) | <0.001 |
|  | Female | 0.73 (0.62-0.87) | <0.001 |
|  | Age | 1.03 (1.03-1.04) | <0.001 |
|  | Ambulance arrival | 3.22 (2.67-3.88) | <0.001 |
| KTAS 5 | Pain group | 0.81 (0.54-1.23) | 0.326 |
|  | Non-medical problem | 0.49 (0.31-0.77) | 0.002 |
|  | Female | 0.67 (0.45-1.00) | 0.048 |
|  | Age | 1.02 (1.01-1.03) | <0.001 |
|  | Ambulance arrival | 3.51 (2.26-5.45) | <0.001 |
