# Supplementary material for: Over-triage occurs when considering the patient's pain in Korean Triage and Acuity Scale (KTAS)
Source: PLoS One. 2019 May 9;14(5):e0216519. doi: 10.1371/journal.pone.0216519 (PMC6508716; doi:10.1371/journal.pone.0216519)
Supplement: S8 Appendix — KTAS, Korean triage and acuity scale; OR, odds ratio; CI, confidence interval; The reference value for complaint category is Gastrointestinal. (DOCX) [file pone.0216519.s008.docx]

| Group | Variable | OR (95% CI) | p-value |
| --- | --- | --- | --- |
| Pain | KTAS 2 | 2.73 (2.21-3.37) | <0.001 |
|  | KTAS 4 | 0.46 (0.38-0.56) | <0.001 |
|  | KTAS 5 | 0.29 (0.18-0.47) | <0.001 |
|  | Female | 0.81 (0.69-0.95) | 0.008 |
|  | Age | 1.02 (1.02-1.03) | <0.001 |
|  | Ambulance arrival | 7.70 (6.51-9.09) | <0.001 |
| Non-pain | KTAS 1 | 171.33 (41.34-710.06) | <0.001 |
|  | KTAS 2 | 4.64 (3.92-5.51) | <0.001 |
|  | KTAS 4 | 0.29 (0.23-0.36) | <0.001 |
|  | KTAS 5 | 0.27 (0.18-0.41) | <0.001 |
|  | Complaint (Respiratory) | 1.41 (1.13-1.78) | 0.003 |
|  | Complaint (Cardiovascular) | 1.75 (1.38-2.23) | <0.001 |
|  | Complaint (Neurological) | 0.58 (0.46-0.74) | <0.001 |
|  | Complaint (Musculoskeletal) | 0.20 (0.14-0.28) | <0.001 |
|  | Complaint (Skin) | 0.29 (0.19-0.42) | <0.001 |
|  | Complaint (General) | 0.48 (0.39-0.61) | <0.001 |
|  | Complaint (Others) | 0.38 (0.29-0.50) | <0.001 |
|  | Female | 0.68 (0.60-0.77) | <0.001 |
|  | Age | 1.02 (1.02-1.03) | <0.001 |
|  | Ambulance arrival | 8.17 (7.11-9.39) | <0.001 |
