# Supplementary material for: Over-triage occurs when considering the patient's pain in Korean Triage and Acuity Scale (KTAS)
Source: PLoS One. 2019 May 9;14(5):e0216519. doi: 10.1371/journal.pone.0216519 (PMC6508716; doi:10.1371/journal.pone.0216519)
Supplement: S9 Appendix — KTAS, Korean triage and acuity scale; OR, odds ratio; CI, confidence interval; The reference value for complaint category is Gastrointestinal. (DOCX) [file pone.0216519.s009.docx]

| Group | Variable | OR (95% CI) | p-value |
| --- | --- | --- | --- |
| Pain | KTAS 2 | 2.07 (1.49-2.89) | <0.001 |
|  | KTAS 4 | 0.53 (0.38-0.72) | <0.001 |
|  | KTAS 5 | 0.37 (0.18-0.74) | 0.005 |
|  | Non-medical problem | 2.07 (1.54-2.79) | <0.001 |
|  | Female | 0.54 (0.42 -0.70) | <0.001 |
|  | Age | 1.02 (1.01-1.02) | <0.001 |
|  | Ambulance arrival | 3.61 (2.78-4.69) | <0.001 |
| Non-pain | KTAS 1 | 11.11 (7.75-15.92) | <0.001 |
|  | KTAS 2 | 2.52 (2.05-3.09) | <0.001 |
|  | KTAS 4 | 0.54 (0.39-0.75) | <0.001 |
|  | KTAS 5 | 0.21 (0.09-0.47) | <0.001 |
|  | Non-medical problem | 3.11 (2.30-4.21) | <0.001 |
|  | Complaint (Respiratory) | 0.87 (0.66-1.15) | 0.328 |
|  | Complaint (Cardiovascular) | 0.28 (0.21-0.39) | <0.001 |
|  | Complaint (Neurological) | 0.36 (0.26-0.49) | <0.001 |
|  | Complaint (Musculoskeletal) | 0.18 (0.11-0.30) | <0.001 |
|  | Complaint (Skin) | 0.09 (0.04-0.19) | <0.001 |
|  | Complaint (General) | 0.71 (0.54-0.94) | 0.015 |
|  | Complaint (Others) | 0.36 (0.25-0.53) | <0.001 |
|  | Female | 0.57 (0.48-0.68) | <0.001 |
|  | Age | 1.01 (1.01-1.02) | <0.001 |
|  | Ambulance arrival | 3.32 (2.77-3.97) | <0.001 |
