# Supplementary material for: Over-triage occurs when considering the patient's pain in Korean Triage and Acuity Scale (KTAS)
Source: PLoS One. 2019 May 9;14(5):e0216519. doi: 10.1371/journal.pone.0216519 (PMC6508716; doi:10.1371/journal.pone.0216519)
Supplement: S10 Appendix — KTAS, Korean triage and acuity scale; OR, odds ratio; CI, confidence interval; The reference value for complaint category is Gastrointestinal. (DOCX) [file pone.0216519.s010.docx]

| Group | Variable | OR (95% CI) | p-value |
| --- | --- | --- | --- |
| Pain | KTAS 2 | 1.08 (0.73-1.58) | 0.706 |
|  | KTAS 4 | 0.46 (0.33-0.65) | <0.001 |
|  | KTAS 5 | 0.20 (0.07-0.53) | 0.001 |
|  | Ambulance arrival | 1.96 (1.46-2.63) | <0.001 |
| Non-pain | KTAS 1 | 1.36 (0.47-3.90) | 0.572 |
|  | KTAS 2 | 1.34 (0.82-2.19) | 0.241 |
|  | KTAS 4 | 0.83 (0.43-1.59) | 0.573 |
|  | KTAS 5 | 0.36 (0.09-1.49) | 0.158 |
|  | Non-medical problem | 1.45 (0.88-2.41) | 0.147 |
|  | Complaint (Respiratory) | 0.67 (0.27-1.68) | 0.394 |
|  | Complaint (Cardiovascular) | 0.68 (0.28-1.62) | 0.383 |
|  | Complaint (Neurological) | 1.15 (0.52-2.53) | 0.728 |
|  | Complaint (Musculoskeletal) | 3.39 (1.49-7.74) | 0.004 |
|  | Complaint (Skin) | 0.42 (0.11-1.71) | 0.228 |
|  | Complaint (General) | 0.71 (0.31-1.66) | 0.433 |
|  | Complaint (Others) | 2.83 (1.35-5.95) | 0.006 |
|  | Ambulance arrival | 2.80 (1.87-4.19) | <0.001 |
