# Supplementary material for: Over-triage occurs when considering the patient's pain in Korean Triage and Acuity Scale (KTAS)
Source: PLoS One. 2019 May 9;14(5):e0216519. doi: 10.1371/journal.pone.0216519 (PMC6508716; doi:10.1371/journal.pone.0216519)
Supplement: S11 Appendix — KTAS, Korean triage and acuity scale; OR, odds ratio; CI, confidence interval; The reference value for complaint category is Gastrointestinal. (DOCX) [file pone.0216519.s011.docx]

| Group | Variable | OR (95% CI) | p-value |
| --- | --- | --- | --- |
| Pain | KTAS 2 | 1.33 (1.13-1.56) | <0.001 |
|  | KTAS 4 | 0.54 (0.47-0.62) | <0.001 |
|  | KTAS 5 | 0.29 (0.21-0.41) | <0.001 |
|  | Non-medical problem | 0.36 (0.30-0.43) | <0.001 |
|  | Female | 0.66 (0.60-0.74) | <0.001 |
|  | Age | 1.03 (1.02-1.03) | <0.001 |
|  | Ambulance arrival | 2.51 (2.20-2.87) | <0.001 |
| Non-pain | KTAS 1 | 3.27 (2.23-4.80) | <0.001 |
|  | KTAS 2 | 1.85 (1.59-2.15) | <0.001 |
|  | KTAS 4 | 0.39 (0.33-0.47) | <0.001 |
|  | KTAS 5 | 0.23 (0.17-0.33) | <0.001 |
|  | Non-medical problem | 0.40 (0.32-0.49) | <0.001 |
|  | Complaint (Respiratory) | 1.05 (0.87-1.28) | 0.607 |
|  | Complaint (Cardiovascular) | 0.44 (0.36-0.54) | <0.001 |
|  | Complaint (Neurological) | 0.34 (0.27-0.41) | <0.001 |
|  | Complaint (Musculoskeletal) | 0.64 (0.49-0.85) | 0.002 |
|  | Complaint (Skin) | 0.24 (0.16-0.36) | <0.001 |
|  | Complaint (General) | 0.97 (0.81-1.16) | 0.742 |
|  | Complaint (Others) | 0.62 (0.50-0.78) | <0.001 |
|  | Female | 0.72 (0.65-0.80) | <0.001 |
|  | Age | 1.03 (1.02-1.03) | <0.001 |
|  | Ambulance arrival | 2.06 (1.83-2.32) | <0.001 |
