# Supplementary material for: Over-triage occurs when considering the patient's pain in Korean Triage and Acuity Scale (KTAS)
Source: PLoS One. 2019 May 9;14(5):e0216519. doi: 10.1371/journal.pone.0216519 (PMC6508716; doi:10.1371/journal.pone.0216519)
Supplement: S12 Appendix — ICU, intensive care unit; KTAS, Korean triage and acuity scale; OR, odds ratio; CI, confidence interval; The reference value for complaint category is Gastrointestinal.; All 490 patients with KTAS 5 in the non-pain group did not admit to ICU, so the statistics were not calculated. (DOCX) [file pone.0216519.s012.docx]

| Group | Variable | OR (95% CI) | p-value |
| --- | --- | --- | --- |
| Pain | KTAS 2 | 3.52 (2.44-5.09) | <0.001 |
|  | KTAS 4 | 0.25 (0.14-0.44) | <0.001 |
|  | KTAS 5 | 0.10 (0.01-0.74) | 0.024 |
|  | Female | 0.46 (0.32-0.65) | <0.001 |
|  | Age | 1.02 (1.01-1.03) | <0.001 |
|  | Ambulance arrival | 3.77 (2.65-5.38) | <0.001 |
| Non-pain | KTAS 1 | 8.30 (5.38-12.78) | <0.001 |
|  | KTAS 2 | 3.96 (3.05-5.15) | <0.001 |
|  | KTAS 4 | 0.32 (0.16-0.64) | 0.001 |
|  | KTAS 5 | Unpredictable | 0.96 |
|  | Complaint (Respiratory) | 2.36 (1.43-3.89) | 0.001 |
|  | Complaint (Cardiovascular) | 3.55 (2.21-5.70) | <0.001 |
|  | Complaint (Neurological) | 4.51 (2.78-7.31) | <0.001 |
|  | Complaint (Musculoskeletal) | 1.33 (0.63-2.80) | 0.458 |
|  | Complaint (Skin) | 0.86 (0.25-3.00) | 0.816 |
|  | Complaint (General) | 0.49 (0.25-0.94) | 0.033 |
|  | Complaint (Others) | 2.22 (1.27-3.87) | 0.005 |
|  | Female | 0.56 (0.45-0.70) | <0.001 |
|  | Age | 1.03 (1.02-1.03) | <0.001 |
|  | Ambulance arrival | 2.78 (2.21-3.49) | <0.001 |
