# Supplementary material for: Over-triage occurs when considering the patient's pain in Korean Triage and Acuity Scale (KTAS)
Source: PLoS One. 2019 May 9;14(5):e0216519. doi: 10.1371/journal.pone.0216519 (PMC6508716; doi:10.1371/journal.pone.0216519)
Supplement: S13 Appendix — KTAS, Korean triage and acuity scale; OR, odds ratio; CI, confidence interval; The reference value for complaint category is Gastrointestinal. All 557 patients with KTAS 5 in the pain group did not expire within 7-day, so the statistics were not calculated. (DOCX) [file pone.0216519.s013.docx]

| Group | Variable | OR (95% CI) | p-value |
| --- | --- | --- | --- |
| Pain | KTAS 2 | 4.34 (1.70-11.09) | 0.002 |
|  | KTAS 4 | 0.35 (0.08-1.59) | 0.174 |
|  | KTAS 5 | Unpredictable | 0.990 |
|  | Female | 0.45 (0.18-1.13) | 0.087 |
|  | Age | 1.06 (1.03-1.09) | <0.001 |
| Non-pain | KTAS 1 | 10.7 (5.71-20.07) | <0.001 |
|  | KTAS 2 | 2.58 (1.54-4.30) | <0.001 |
|  | KTAS 4 | 0.18 (0.03-1.36) | 0.097 |
|  | KTAS 5 | 0.59 (0.08-4.39) | 0.605 |
|  | Non-medical problem | 0.28 (0.09-0.91) | 0.035 |
|  | Female | 0.62 (0.39-0.97) | 0.037 |
|  | Age | 1.03 (1.01-1.04) | <0.001 |
|  | Ambulance arrival | 4.97 (2.81-8.81) | <0.001 |
