# Supplementary material for: Over-triage occurs when considering the patient's pain in Korean Triage and Acuity Scale (KTAS)
Source: PLoS One. 2019 May 9;14(5):e0216519. doi: 10.1371/journal.pone.0216519 (PMC6508716; doi:10.1371/journal.pone.0216519)
Supplement: S14 Appendix — KTAS, Korean triage and acuity scale; OR, odds ratio; CI, confidence interval; The reference value for complaint category is Gastrointestinal.; All 167 patients in the non-pain group with KTAS 1 were severe, so the statistics were not calculated. (DOCX) [file pone.0216519.s014.docx]

| Group | Variable | OR (95% CI) | p-value |
| --- | --- | --- | --- |
| Pain | KTAS 2 | 1.52 (1.30-1.78) | <0.001 |
|  | KTAS 4 | 0.51 (0.44-0.58) | <0.001 |
|  | KTAS 5 | 0.27 (0.20-0.37) | <0.001 |
|  | Non-medical problem | 0.50 (0.43-0.58) | <0.001 |
|  | Female | 0.67 (0.61-0.74) | <0.001 |
|  | Age | 1.03 (1.02-1.03) | <0.001 |
|  | Ambulance arrival | 4.07 (3.57-4.64) | <0.001 |
| Non-pain | KTAS 1 | Unpredictable | 0.923 |
|  | KTAS 2 | 2.98 (2.51-3.55) | <0.001 |
|  | KTAS 4 | 0.35 (0.30-0.42) | <0.001 |
|  | KTAS 5 | 0.24 (0.17-0.32) | <0.001 |
|  | Non-medical problem | 0.62 (0.51-0.75) | <0.001 |
|  | Complaint (Respiratory) | 1.33 (1.08-1.65) | 0.008 |
|  | Complaint (Cardiovascular) | 1.22 (0.97-1.54) | 0.092 |
|  | Complaint (Neurological) | 0.45 (0.37-0.56) | <0.001 |
|  | Complaint (Musculoskeletal) | 0.41 (0.31-0.54) | <0.001 |
|  | Complaint (Skin) | 0.26 (0.19-0.37) | <0.001 |
|  | Complaint (General) | 0.85 (0.71-1.03) | 0.099 |
|  | Complaint (Others) | 0.49 (0.39-0.62) | <0.001 |
|  | Female | 0.69 (0.61-0.77) | <0.001 |
|  | Age | 1.03 (1.02-1.03) | <0.001 |
|  | Ambulance arrival | 5.32 (4.64-6.10) | <0.001 |
